# Ultraviolet Light‐Assisted Decontamination of Chemical Warfare Agent Simulant 2‐Chloroethyl Phenyl Sulfide on Metal‐Loaded TiO_2_/Ti Surfaces

**DOI:** 10.1002/open.202300246

**Published:** 2024-02-20

**Authors:** Hye Ji. Jang, Gaeun Yun, Huieun Shim, Seon Young Hwang, So Young Kim, Jeongkwon Kim, Heesoo Jung, Mohammad Mansoob Khan, Youngku Sohn

**Affiliations:** ^1^ Department of Chemistry Chungnam National University Daejeon 34134 Republic of Korea; ^2^ Agency for Defense Development (ADD) Daejeon 34186 Republic of Korea; ^3^ Chemical Sciences Faculty of Science Universiti Brunei Darussalam Jalan Tungku Link Gadong BE 1410 Brunei Darussalam

**Keywords:** Chemical warfare agent, 2-chloroethyl phenyl sulfide, UV photodecontamination, Secondary byproducts, TiO_2_/Ti, Overlaid Au

## Abstract

The application of ultraviolet (UV) light for the decontamination of chemical warfare agents (CWAs) has gained recognition as an effective method, especially for treating hard‐to‐reach areas where wet chemical methods are impractical. In this study, TiO_2_/Ti was employed as a model catalyst, which was contaminated with 2‐chloroethyl phenyl sulfide (CEPS), and subjected to photocatalytic decontamination using both UVB and UVC light. Additionally, photocatalytic decontamination efficiency by introducing Au, Pt, and Cu onto the TiO_2_/Ti surface was explored. During the photodecomposition process under UVC light, at least eight distinct secondary byproducts were identified. It was observed that the introduction of overlayer metals did not significantly enhance the photodecomposition under UVC light instead overlaid Au exhibited substantially improved activity under UVB light. Whereas, photodecomposition process under UVB light, only five secondary products were detected, including novel compounds with sulfoxide and sulfone functional groups. This novel study offers valuable insights into the generation of secondary products and sheds light on the roles of overlayer metals and photon wavelength in the photodecontamination process of CWA.

## Graphical abstract

UV light emerges as a potent decontaminant for chemical warfare agents (CWAs). Utilizing TiO_2_/Ti as a catalyst, this study investigates UV photocatalytic decontamination efficiency against 2‐chloroethyl phenyl sulfide. Introduction of Au, Pt, and Cu onto TiO_2_/Ti surface is explored. UVC light reveals eight secondary byproducts, while Au overlay notably boosts activity under UVB, uncovering valuable insights for CWA decontamination.

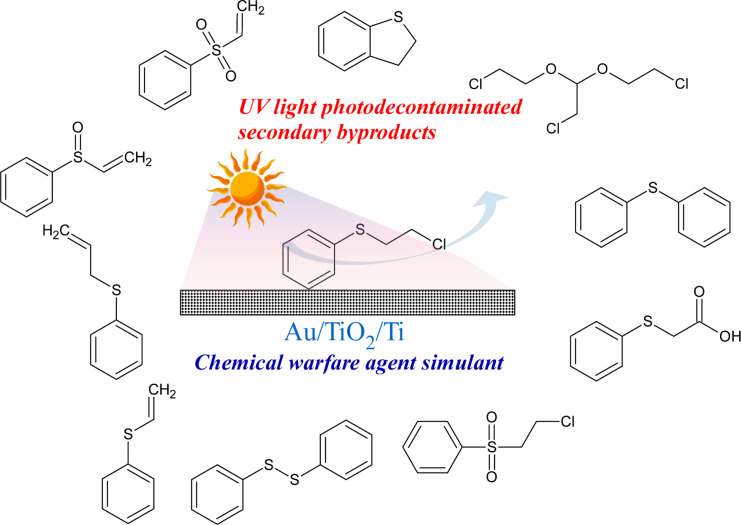

## 1 Introduction

Chemical warfare agents (CWAs) pose a serious threat to human security worldwide. Extensive laboratory research has been conducted to develop detoxification methods for chemical warfare agents (CWAs) using a CWA simulant in targeted areas.[^1^, ^2^, ^3^, ^4^, ^5^, ^6^, ^7^, ^8^, ^9^, ^10^] Various techniques, including adsorption, wet, and dry decontamination methods, have been explored. 2‐chloroethyl phenyl sulfide (CEPS) and 2‐chloroethyl ethyl sulfide (CEES) are commonly studied CWA simulants due to their resemblance to highly toxic agents such as sulfur mustard and VX (a thiophosphonate ester).[^11^, ^12^, ^13^, ^14^, ^15^, ^16^, ^17^, ^18^, ^19^, ^20^, ^21^, ^22^, ^23^, ^24^, ^25^, ^26^, ^27^, ^28^, ^29^, ^30^]

Various strategic approaches were employed to achieve the desired objective. These strategies encompass material‐based techniques such as adsorption and catalyst utilization, with a wide range of demonstrated materials, including metal‐organic frameworks (MOFs),[^7^, ^12^] TiO_2_/Mg(OH)_2_,^[13]^ reactive polymers and organic materials,[^14^, ^15^] Zr(OH)_4_/fiber composites,^[16]^ granular UiO‐66‐NH_2_ metal‐organic gels,^[17]^ molybdate‐based ionic liquids/H_2_O_2_ systems,^[18]^ Au‐incorporated TiO_2_,^[19]^ and Pb‐MCM‐41/ZnNiO_2_.^[20]^ For examples, Gorden et al. investigated the decontamination of 2‐chloroethyl ethyl sulfide (2‐CEES) on Au/TiO_2_ under visible light (λ>400 nm) and in both anaerobic and aerobic conditions.^[19]^ They observed photothermal desorption of 2‐CEES under anaerobic conditions, whereas a hydrolysis reaction occurred under aerobic conditions, with no visible light photocatalytic effect reported. Giles et al. synthesized various TiO_2_‐based nanomaterials and utilized them for the photodecontamination (λ>400 nm) of 2‐CEES under ambient conditions. They highlighted that reactivity was significantly influenced by surface and structural factors, specifically related to active sites, polarity, surface area, and light absorption.^[25]^

Decontamination methods encompass wet chemical techniques,^[3]^ high‐power lasers,^[11]^ UV light,^[31]^ plasma,^[23]^ and microwave technology.^[24]^ Ultraviolet and laser light have been utilized for the photodecontamination of the CWA simulant. In a related study, Lee et al. employed high‐power lasers with wavelengths of 266 nm and 355 nm to photo‐decontaminate CEPS on quartz glass. They observed a wavelength‐dependent effect and identified four distinct decomposition products, with a prominent one being diphenyl disulfide (C_12_H_10_S_2_).^[11]^ The specific methodology used can influence the composition of the decomposition products, and further investigation of secondary products is crucial for devising safer treatment strategies.

Driven by our goal, authors delve into the byproducts produced during the photocatalytic decontamination of the original CWA simulant molecule. CEPS was chosen for the study due to its relatively lower level of prior research compared to CEES.[^11^, ^12^, ^13^, ^14^, ^15^, ^16^, ^17^, ^18^, ^19^, ^20^, ^21^, ^22^, ^23^, ^24^, ^25^, ^26^, ^27^, ^28^, ^29^, ^30^] The introduction of both UVC and UVB lights was based on the hypothesis that decontamination could commence through the direct cleavage of strong and weak chemical bonds or via an indirect charge transfer process. TiO_2_ was selected as the model catalyst material.[^25^, ^33^, ^34^] Additionally, Au, Pt, and Cu were loaded onto TiO_2_ to investigate the roles of these metals under both UVB and UVC lights. This novel research aims to provide a deeper understanding of the photocatalytic decontamination of CWA molecules in diverse environmental conditions.

## Experimental Section

**CEPS contaminated on TiO_2_/Ti sheet**. The CWA molecule of 2‐chloroethyl phenyl sulfide (CEPS, 98 %, Sigma–Aldrich) was used as received without any additional purification. Titanium (Ti) sheets (Grade 2, 0.1 mm thick, sourced from Aliexpress) were cut into square pieces measuring 30 mm in length and 30 mm in width. These pieces underwent a thorough cleaning process involving acetone and isopropyl alcohol. Subsequently, the sheets were subjected to ozone cleaning and then thermally treated at 700 °C for 1 h in a conventional convection furnace to create a fully oxidized Ti surface, referred to as TiO_2_/Ti sheet.^[32]^ To sputter‐deposit metal layers (Au, Cu, and Pt) onto the TiO_2_/Ti substrate, a SPT‐20 ion sputter coater from COXEM Co., Korea was employed, with an ionization current of 3 mA for a specified duration. The resulting samples were denoted as M/TiO_2_/Ti. To conduct photocatalytic decontamination tests in a closed system under UV light, a sealed stainless steel reactor was fabricated. CEPS liquid (10 μL) was dispensed onto a TiO_2_/Ti sheet, which was then placed inside a 40‐cm^3^ stainless reactor and then sealed for leak‐free, but under atmospheric air (with O_2_ and H_2_O) condition. The reactor cover featured a quartz window (with a 45 mm diameter) to allow for light irradiation onto the sample, as previously described in the literature.[^31^, ^32^]

**UV photocatalytic decontamination and gas chromatography (GC)‐mass analysis**. For the photocatalytic decontamination tests lasting 5 h under UV lamps, the reactor, equipped with a quartz window, was exposed to 15 W UVB (290–320 nm) and UVC (200–290 nm) lamps (measuring 2.5 cm in diameter and 43.6 cm in length). These lamps had power densities of 5.38 and 5.94 mW ⋅ cm^−2^, respectively. After UV irradiation for 5 h, the liquid residues were extracted from the TiO_2_/Ti using 1 mL of dichloromethane, followed by adequate mixing, filtration, and a 10‐fold dilution. Subsequently, a 1 μL aliquot of the diluted sample was introduced for analysis. The liquid products (or secondary byproducts) were examined using a Shimadzu GCMS‐QP2010 (Shimadzu, Kyoto, Japan) at the CNU Core Facility. An Agilent J&W HP‐5 ms capillary column (30 m×0.25 mm×0.25 μm) was employed with helium as the carrier gas.

**Characterization of the uncontaminated and contaminated surfaces**. TiO_2_/Ti was subjected to various characterizations to investigate its surface morphology, surface oxidation state, and crystal phase. The surface morphologies of bare TiO_2_/Ti were analyzed through scanning electron microscopy (SEM), utilizing a Hitachi S‐4800 instrument with an acceleration voltage of 10 keV. The crystal phase of TiO_2_/Ti was determined using a MiniFlex II X‐ray diffractometer (Rigaku Corp., CNU Chemistry Core Facility Center) equipped with a Cu Kα X‐ray source (40 kV and 30 mA). To confirm the crystallinity of TiO_2_/Ti, Raman spectra were collected using a Horiba Jobin Yvon LabRAM HR‐800 UV–Visible‐NIR Raman spectrometer with a 514 nm laser line. Liquid residues after the photodecomposition of CEPS on TiO_2_/Ti sheets and M/TiO_2_/Ti sheets (M=Au, Cu, and Pt) were analyzed using a FT‐IR spectrometer (Nicolet iS10, Thermo Fisher Scientific). The reflectance UV–visible absorption spectra of TiO_2_/Ti sheets were acquired using a double‐beam UV–visible absorption spectrometer (Neosys‐2000, Scinco Co., Ltd.). For TiO_2_/Ti and Au/TiO_2_/Ti, oxidation states and the presence of overlayer Au were assessed through X‐ray photoelectron spectroscopy (XPS). This analysis was performed using a Thermo Scientific K‐Alpha^+^ XP spectrometer equipped with a hemispherical energy analyzer and a monochromated Al Kα X‐ray source (1486.7 eV). For XPS test sample preparation, as the sample surface was wet, we washed it with dichloromethane, following the procedure mentioned above for GC. Subsequently, we dried the samples in a hood overnight using gentle air blowing

## 2 Results and Discussion

**Figure 1:**
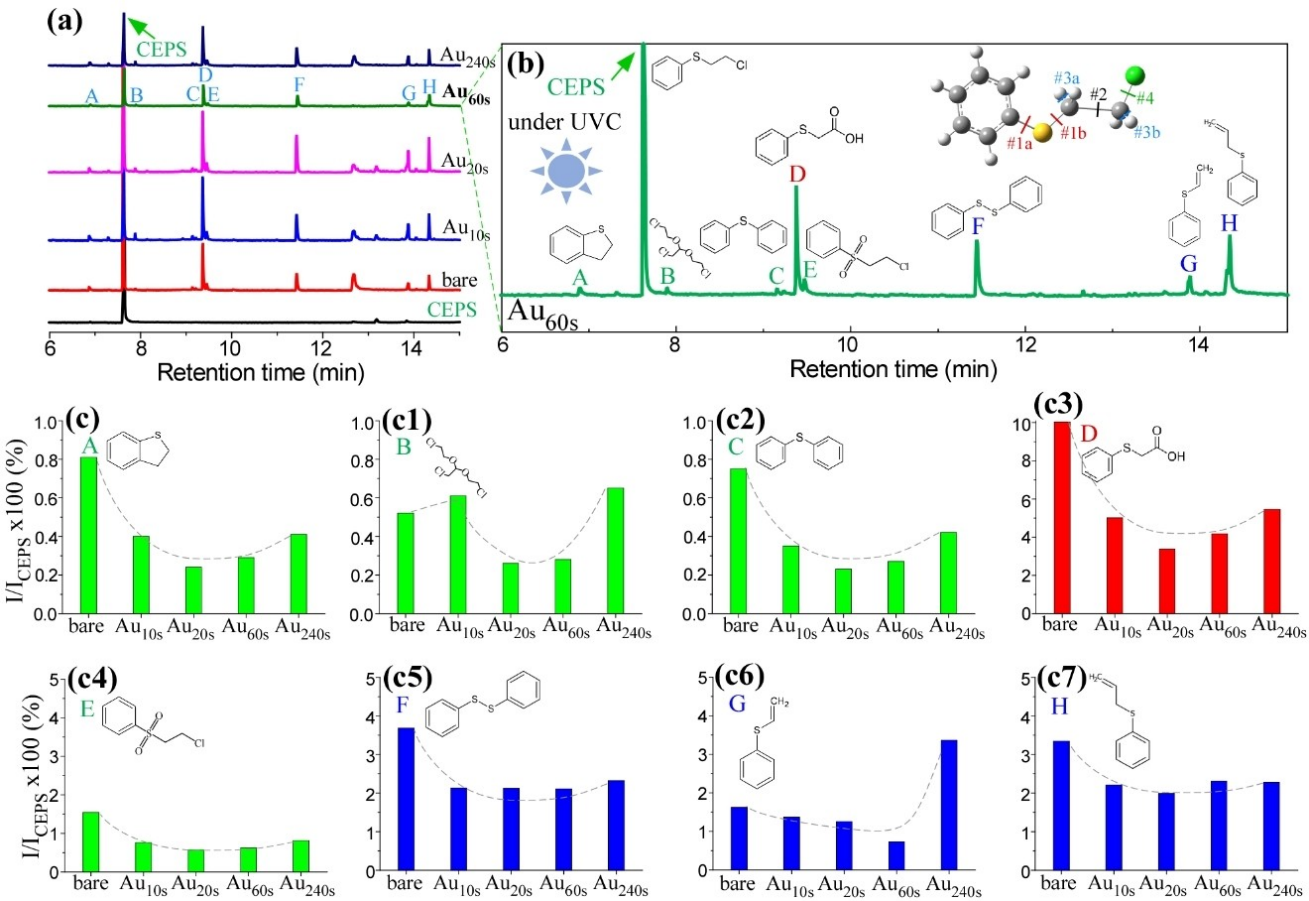
GC‐MS profiles of CEPS and after photocatalytic decontamination over bare TiO_2_/Ti and Au/TiO_2_/Ti with Au deposition time (a). Selected GC‐MS profile for Au_60s_/TiO_2_/Ti showing decontaminated byproducts (A to H), and inset shows CEPS showing plausible bond breakages (b). Relative intensity of the product compared with CEPS peak, I/I_CEPS_×100 (%) (c to c7).

**Photocatalytic decontamination of CEPS under UVC light**. Decontamination tests were conducted for 2‐chloroethyl phenyl sulfide (CEPS) on both bare TiO_2_/Ti and Au/TiO_2_/Ti with varying Au deposition times under UVC (200–290 nm) irradiation for a duration of 5 h. Figure 1 presents the GC‐MS profiles illustrating the composition of residual liquid products after the experiments. Prior to photocatalytic decontamination, a prominent peak appeared at a retention time of 7.6 minutes, corresponding to the CEPS molecule. Other peaks were attributed to residual impurities, which were not discussed further. During UVC irradiation, several new peaks emerged with varying intensities. By cross‐referencing with a mass library database, a minimum of eight distinct species were identified, each displaying different intensities. The chemical structures of these products are depicted in Figure 1b and labeled from A to F. The secondary byproducts included A: 2,3‐dihydro benzothiophene (C_8_H_8_S), B: 2‐chloro‐1,1‐bis(2‐chloroethoxy)ethane (C_6_H_11_Cl_3_O_2_), C: diphenyl sulfide (C_12_H_10_S), D: (phenylthio)acetic acid (C_8_H_8_O_2_S), E: 2‐chloroethyl phenyl sulfone (C_8_H_9_ClO_2_S), F: diphenyl disulfide (C_12_H_10_S_2_), G: phenyl vinyl sulfide (C_8_H_8_S), and H: allyl phenyl sulfide (C_9_H_10_S). The GC peaks of D, F, G, and H were found to be stronger than the others. In the GC profiles, the CEPS after photodecontamination exhibited a larger peak compared to the others. This could be attributed to a high contamination amount of CEPS (with 10 μL) on the catalyst surface, which has an area of 3 cm×3 cm.

**Scheme 1:**
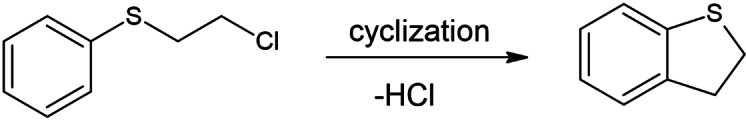
Reaction mechanism for A (2,3‐dihydro benzothiophene).

**Scheme 2:**
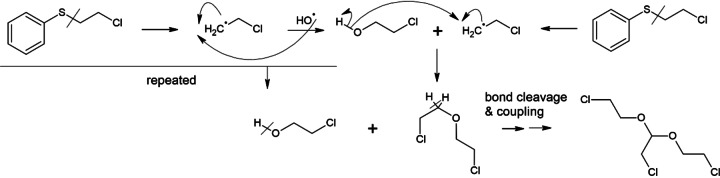
Reaction mechanism for B (2‐chloro‐1,1‐bis(2‐chloroethoxy)ethane).

**Scheme 3:**
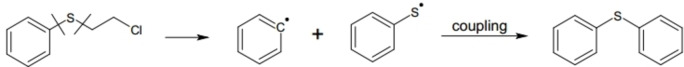
Reaction mechanism for C (diphenyl sulfide).

**Scheme 4:**
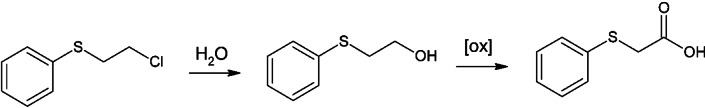
Reaction mechanism for D ((phenylthio)acetic acid).

**Scheme 5:**
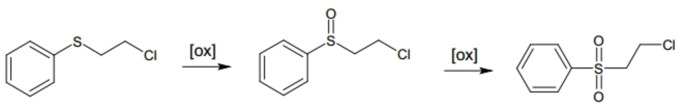
Reaction mechanism for E (2‐chloroethyl phenyl sulfone).

**Scheme 6:**
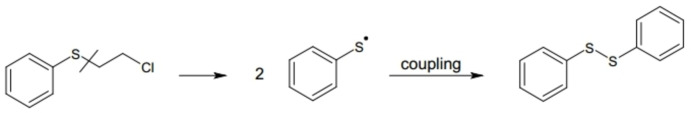
Reaction mechanism for F (diphenyl disulfide).

**Scheme 7:**
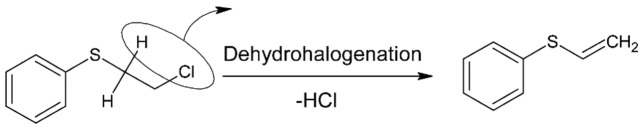
Reaction mechanism for G (phenyl vinyl sulfide)

**Scheme 8:**
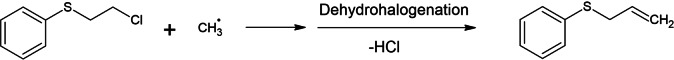
Reaction mechanism for H (allyl phenyl sulfide).

In the CEPS molecule, five bonds are likely to be broken under UVC irradiation, denoted as #1a (C_ph_−S), #1b (S−CH_2_), #2 (C−C), #3a/#3b (C−H of CH_2_), and #4 (C−Cl). For the sake of clarity in the current discussion, reference to the C−H and C−C bonds within the phenyl (=Ph) group have been omitted. In the case of the A product, 2,3‐dihydrobenzothiophene in Scheme 1, the initial event involved the breaking of the C−Cl bond, leading to the formation of Ph−S−CH_2_CH_2_⋅, followed by a subsequent cyclization.^[35]^ For the B product with more complicated processes in Scheme 2, the breaking of the C−S bond (#1b) led to the formation of ⋅CH_2_CH_2_Cl, which was subsequently oxidized to ⋅OCH_2_CH_2_Cl. This oxidation process and its coupling reactions may result in the formation of the product hinted at in the literature.^[28]^ In the case of the C product in Scheme 3, the breaking of two C−S bonds (#1a and #1b) generated two radical species, Ph⋅, and Ph−S⋅, which then coupled to produce diphenyl sulfide.^[28]^ Regarding the D product in Scheme 4, the C−Cl bond (#2) was broken through a hydrolysis reaction,^[21]^ resulting in the creation of a primary alcohol. This primary alcohol was further oxidized to form a carboxylic acid.^[36]^ For the E product in Scheme 5, an oxidation reaction of CEPS with reactive oxygen species, such as singlet oxygen and superoxide radicals, occurred, leading to the formation of a sulfone, as depicted below.[^21^, ^26^, ^37^] In the case of the F product in Scheme 6, diphenyl disulfide (C_12_H_10_S_2_) was detected at a retention time of 11.4 min, the initial step involved the cleavage of the C−S bond, followed by a subsequent coupling reaction between two radical Ph−S⋅ species, resulting in the formation of Ph−S−S−Ph.[^11^, ^38^] When examining CEPS under UV laser irradiation, Lee et al. reported that the predominant product among the four byproducts, which included ethenylthiobenzene (C_8_H_8_OS), diphenyl disulfide (C_12_H_10_S_2_), phenyl vinyl sulfide (C_8_H_8_S), and (3,3,5,5‐tetramethylcyclohex‐1‐en‐1‐yl)thiobenzene (C_16_H_20_S), was found to be diphenyl disulfide.^[11]^ In the case of phenyl vinyl sulfide (G) in Scheme 7, the breaking of both C−H and C−Cl bonds led to the elimination of HCl, resulting in the product through a dehydrohalogenation reaction.[^13^, ^21^] This particular product has been documented in the literature.^[13]^ For instance, Stastny et al. utilized TiO_2_/Mg(OH)_2_ composites for the degradation of CEPS, where they identified phenyl vinyl sulfide as a major degradation product.^[13]^ This product has also been reported in the context of CEPS decontamination over‐dispersed ZnO nanoparticles under light‐free conditions.^[22]^ Furthermore, a hydrolysis product of hydroxyethyl phenyl sulfide was additionally observed. For the H product in Scheme 8, ⋅CH_3_ may participate in the elimination reaction to form the product.

Figures 1c to 1c7 depict the GC peak intensities after normalizing CEPS intensity to 100 for both bare TiO_2_/Ti and Au/TiO_2_/Ti with varying Au deposition times. The relative ratio can be represented as I/I_CEPS_×100 (%). Under UVC conditions, a common trend emerged where activity initially decreased upon Au deposition compared to bare TiO_2_/Ti. However, with further Au deposition, activity subsequently increased. Among these scenarios, Au_20s_/TiO_2_/Ti exhibited the lowest activity for products A to E. Similar trends were observed for products F to H, with one exception: for product G, Au_240s_/TiO_2_/Ti displayed the highest activity in the formation of phenyl vinyl sulfide. This suggests that the presence of Au may facilitate the elimination of HCl, leading to the increased production of phenyl vinyl sulfide.^[39]^

**Figure 2:**
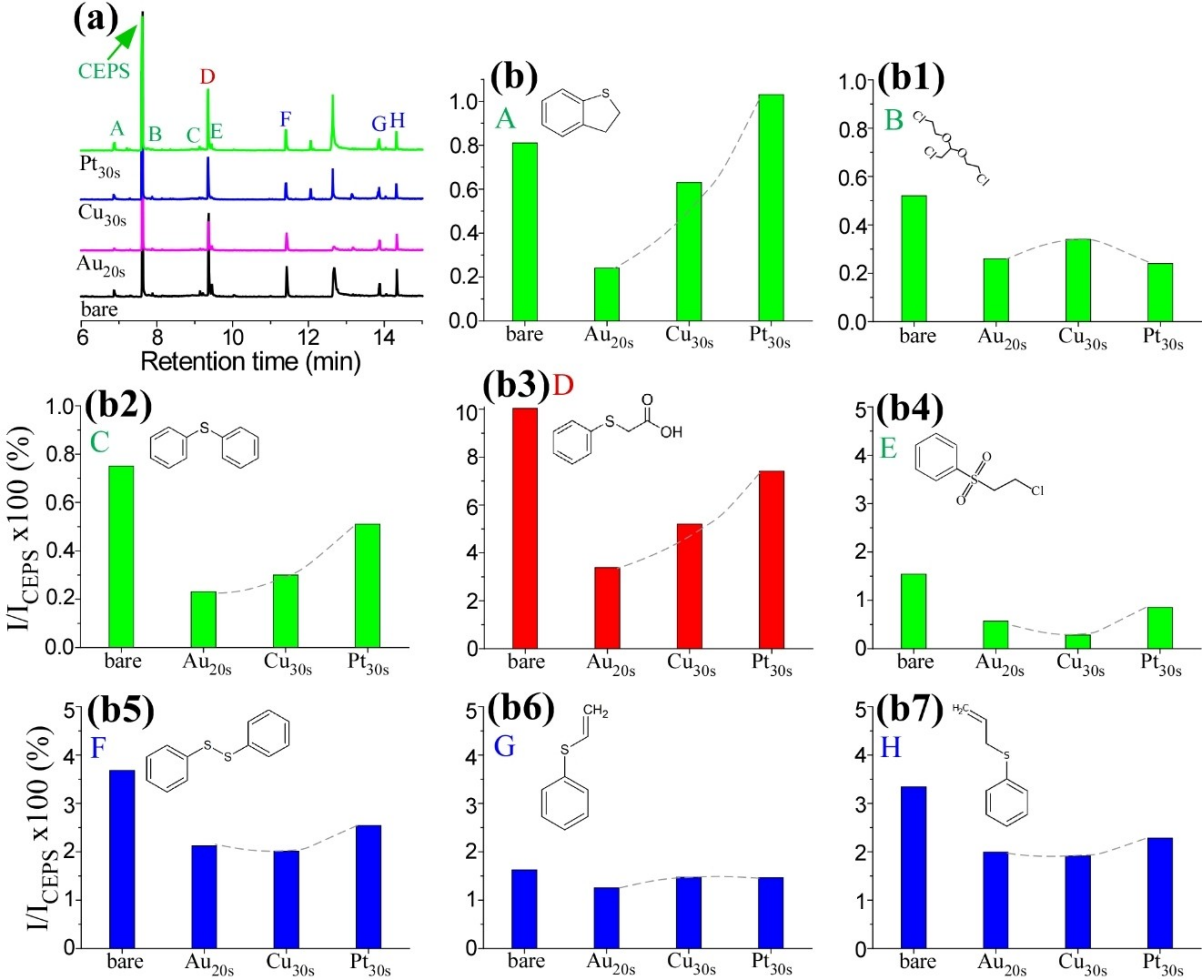
GC‐MS profiles of CEPS after photocatalytic decontamination over bare TiO_2_/Ti, Au_20s_/TiO_2_/Ti, Cu_30s_/TiO_2_/Ti, and Pt_30s_/TiO_2_/Ti (a). Relative intensities of the secondary byproducts compared with the CEPS peak, I/I_CEPS_×100 (%) for the four different catalysts under UVC conditions (b to b7).

**Photocatalytic decontamination of CEPS over different metal‐loaded TiO_2_/Ti**. A brief photocatalytic decomposition test on TiO_2_/Ti with different overlayer metals including Au, Cu, and Pt (as shown in Figure 2a) was conducted. A common observation was that the catalytic activities of M/TiO_2_/Ti (M=Au, Cu, and Pt) were generally lower when compared to bare TiO_2_/Ti, except in the case of the product A (2,3‐dihydrobenzothiophene). Among the overlayer metals, Pt/TiO_2_/Ti displayed the highest activity in the formation of product A. As mentioned earlier, product A results from a cyclization reaction, and these results suggest that the Pt overlayer played a more significant role in initiating the cyclic process.^[40]^ In the case of products A, C, and D, the activity exhibited the order of Au<Cu<Pt, indicating a strong dependence on the choice of overlayer metal. For other products, the activity variations were not as dramatic as observed for these three products. In the case of products B and G, Cu/TiO_2_/Ti showed somewhat higher activity than Au/TiO_2_/Ti and Pt/TiO_2_/Ti, whereas for products E, F, and H, Cu/TiO_2_/Ti exhibited poorer activity compared to the other two.

**Figure 3:**
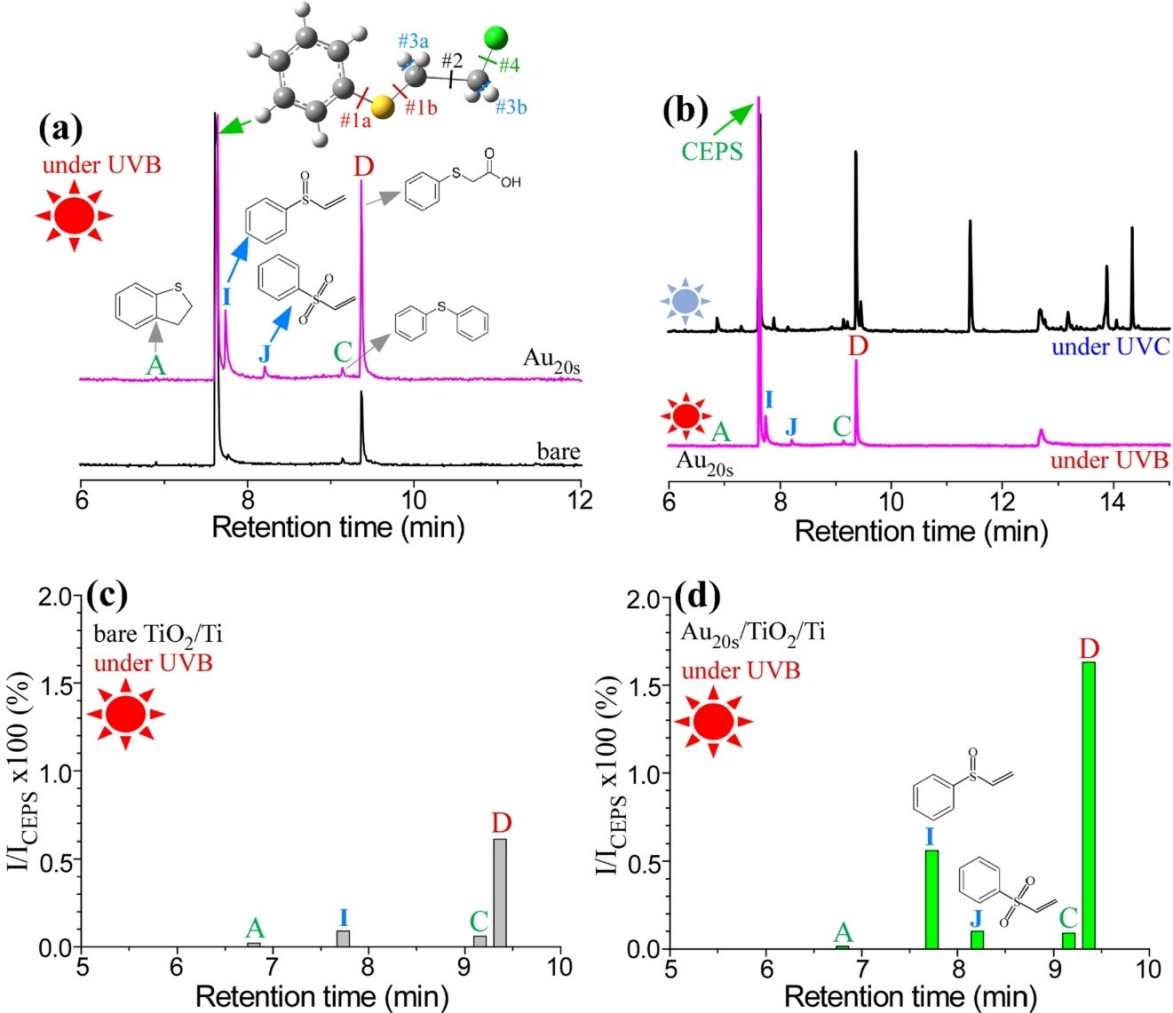
GC‐MS profiles of CEPS after photocatalytic decontamination over bare TiO_2_/Ti and Au_20s_/TiO_2_/Ti under UVB condition (a). GC‐MS profiles of CEPS after photodecontamination over Au_20s_/TiO_2_/Ti under UVC and UVB conditions (b). Relative intensities of the secondary byproducts compared with the CEPS peak, I/I_CEPS_×100 (%) over bare TiO_2_/Ti and Au_20s_/TiO_2_/Ti under UVB condition (c and d, respectively).

**Photocatalytic decontamination of CEPS under UVB light**. Photocatalytic decomposition experiments were conducted under UVB (290–320 nm) using bare TiO_2_/Ti and Au_20s_/TiO_2_/Ti to examine the influence of photon wavelength and the overlayer Au. As discussed earlier, the Au_20s_/TiO_2_/Ti exhibited significantly lower activity for CEPS decomposition under UVC irradiation compared to bare TiO_2_/Ti. However, in Figure 3a, the Au_20s_/TiO_2_/Ti displayed a much higher production of decomposition products than bare TiO_2_/Ti. Products A, C, and D were observed under both UVB and UVC conditions in the GC profiles (Figure 1a and 1b). However, certain byproducts detected under UVC were entirely absent under UVB conditions (Figure 3b). Notably, under UVB irradiation, two different products were newly identified with retention times of 7.7 minutes and 8.2 minutes. These were identified as I=(ethenylsulfinyl)benzene (C_8_H_8_OS) and J=phenyl vinyl sulfone (C_8_H_8_O_2_S) based on mass library database information.

**Scheme 9:**
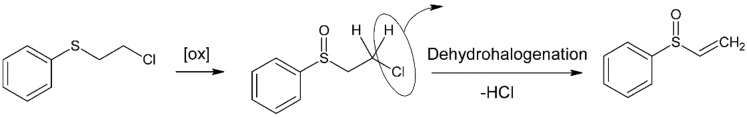
Reaction mechanism for I ((ethenylsulfinyl)benzene).

**Scheme 10:**
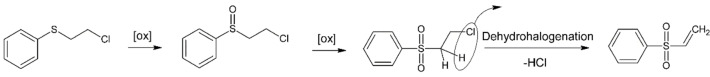
Reaction mechanism for J (phenyl vinyl sulfone).

The relative ratios, expressed as I/I_CEPS_×100 (%), were analyzed for both bare TiO_2_/Ti and Au_20s_/TiO_2_/Ti, as shown in Figures 3c and 3d, respectively. Clearly, Au_20s_/TiO_2_/Ti exhibited significantly higher activity in degrading CEPS under UVB irradiation. Figure 3c displays the detection of four byproducts (A, I, C, and D) over bare TiO_2_/Ti. However, when Au was introduced, an additional product with a sulfone group (J) emerged over Au_20s_/TiO_2_/Ti. Notably, the products I and J were significantly more pronounced in the presence of Au, compared to the C and D products. This observation suggests that O_2_ primarily adsorbed to the periphery of Au and TiO_2_ and played a more active role in oxidizing sulfide to produce sulfoxide and sulfone. These two byproducts were characterized by the presence of functional groups, sulfoxide (S=O) and sulfone (O=S=O),[^26^, ^28^, ^37^] indicating the oxidation of sulfide (R−S−R′) in the presence of reactive oxygen species like ^1^O_2_ and O_2_⋅^−^, as discussed further below. The production mechanism (with oxidation and dehydrohalogenation reactions) is depicted in Schemes 9 and 10. Wang et al. also reported the formation of sulfoxide (major) and sulfone (minor) byproducts after the photocatalytic decontamination of CEES under a blue LED,^[26]^ which is in good agreement with the findings of this study.

The GC peak intensity followed the order of A<C<J≪I≪D. Based on the observed byproducts, it is plausible that the initial bond breakages occurred in the C−S and C−Cl bonds. Bond energy is known to follow the order of C−S (#1a and #1b)<C−Cl (#4)<C−C (#2)<C−H (#3). Consequently, the C−S bond (#1a and #1b) was expected to be the most easily broken under UVB irradiation. The breaking of the C−S bond was expected to result in the formation of the C product, which exhibited a weaker peak compared to others (I, J, and D). These products were found to be free of chlorine (Cl), indicating that C−Cl bond breakage contributed more to the formation of these products. The more pronounced breakage of the C−Cl bond could plausibly be attributed to the presence of the Au overlayer. For instance, the strong interaction between Au and Cl may weaken the C−Cl bond.^[41]^

**Figure 4:**
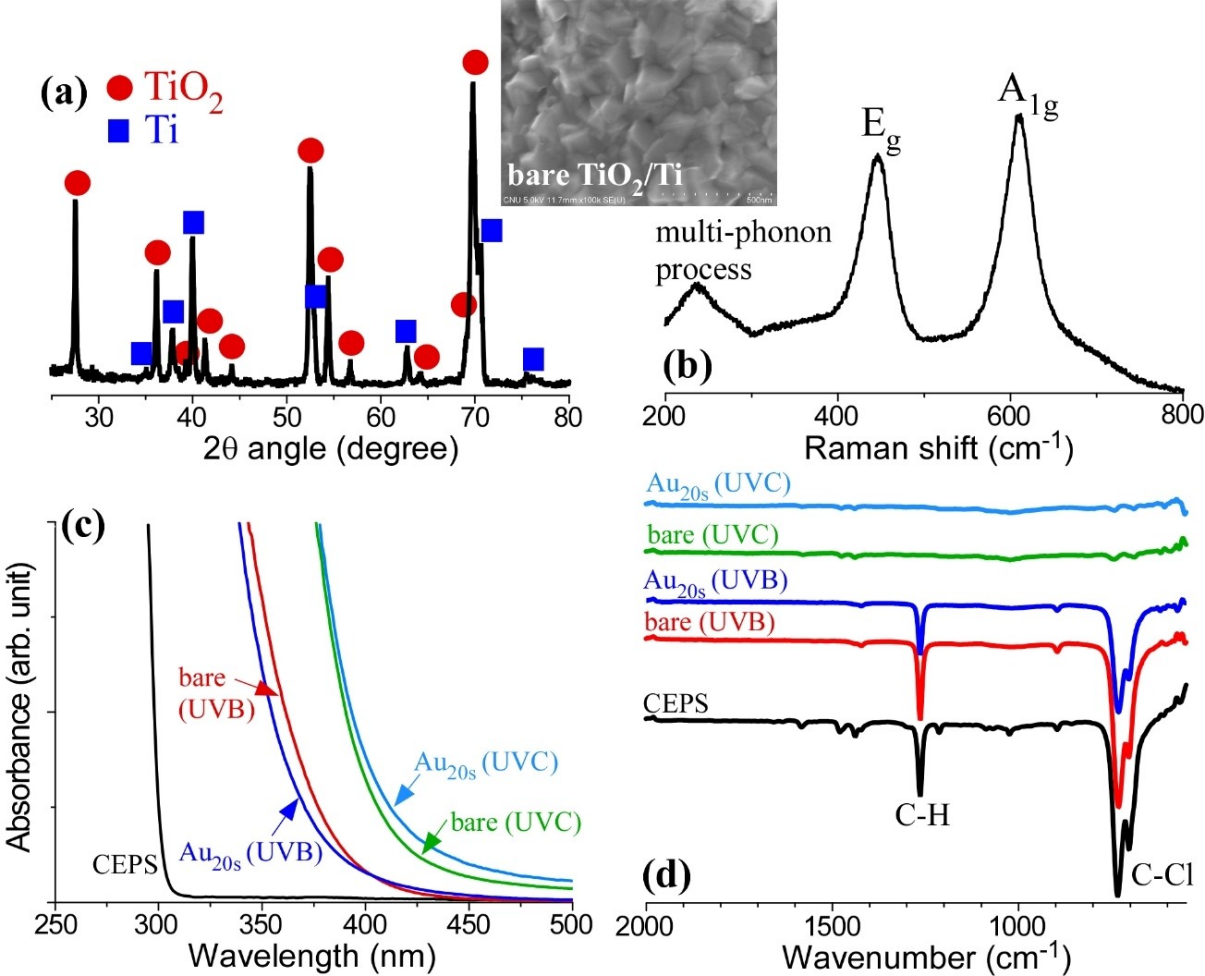
XRD pattern of TiO_2_/Ti (a). Raman spectra and SEM image (in the inset) of TiO_2_/Ti (b). UV–vis absorption spectra of CEPS before and after photocatalytic decontamination over bare TiO_2_/Ti and Au_20s_/TiO_2_/Ti under UVC and UVB light conditions (c). Corresponding FT‐IR spectra of CEPS before and after photodecontamination over bare TiO_2_/Ti and Au_20s_/TiO_2_/Ti under UVC and UVB light conditions (d).

**Characterization of TiO_2_/Ti and CEPS before and after photocatalytic decontamination**. The prepared TiO_2_/Ti was thoroughly examined using XRD, SEM, and Raman analyses, all of which confirmed the presence and formation of TiO_2_ on the Ti support. The X‐ray diffraction (XRD) analysis of TiO_2_/Ti revealed two distinct diffraction patterns as shown in Figure 4a: one corresponding to the Ti support and the other to TiO_2_ grown on the Ti substrate. The XRD peaks (represented by closed red circles) at 2θ angles of 27°, 36°, 39°, 41°, 44°, 54°, 57°, 63°, 64°, 69°, and 70° were identified as the (110), (011), (020), (111), (120), (121), (220), (002), (130), (031), and (112) crystal planes of tetragonal rutile TiO_2_.[^32^, ^42^] The other XRD peaks (represented by closed squares) were observed at 2θ angles of 35°, 38°, 40°, 53°, 63°, 76°, and 77°, and were attributed to the (010), (002), (011), (012), (110), (013), (112), and (021) planes of hexagonal metallic Ti. In Figure 4b, Raman signals were detected at 445 cm^−1^ and 610 cm^−1^, which were attributed to the E_g_ and A_1g_ symmetry vibrational modes, respectively, in rutile TiO_2_ with a D_4h_ point group.[^43^, ^44^] Additionally, a broad band at 240 cm^−1^ was assigned to the 2nd‐order (or multi‐phonon) Raman scattering. This outcome affirmed the high crystallinity of the TiO_2_ grown on the Ti support. The corresponding SEM image in the inset resembled a rocky texture.

UV–visible absorption spectra were acquired (Figure 4c) for CEPS both before and after photocatalytic decontamination on bare TiO_2_/Ti and Au_20s_/TiO_2_/Ti under both UVC and UVB light conditions. In the case of pristine CEPS, strong absorption was observed below 300 nm, consistent with previous literature findings.^[11]^ Post photocatalytic decontamination, the absorption range expanded into the visible spectrum, indicating the formation of products with different structures capable of absorbing visible light. The absorption range was notably broader under UVC conditions, suggesting the generation of a more diverse array of byproducts, in alignment with the findings presented in Figure 3b. Conversely, UVB conditions exhibited a narrower UV–visible absorption range.

The FT‐IR spectra corroborated the UV–visible absorption results depicted in Figure 4d. In the FT‐IR spectrum of pristine CEPS, strong C−H and C−Cl vibrational modes were evident.^[11]^ These peaks exhibited reduced intensity under UVB irradiation and further dramatic attenuation following UVC irradiation. These observations align well with the findings from the GC‐mass results presented in Figure 3b and the UV–visible absorption results in Figure 4c.

**Figure 5:**
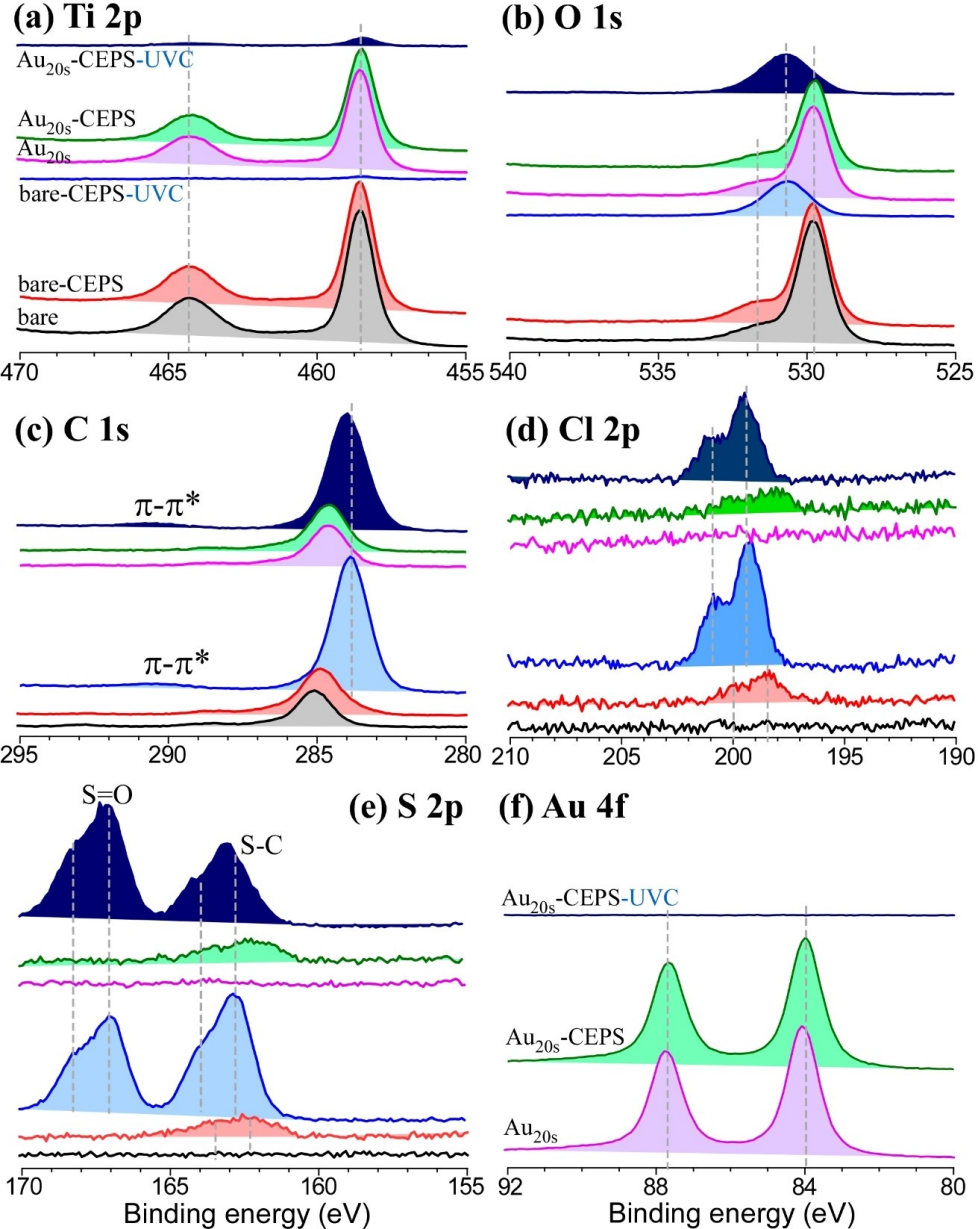
Ti 2p (a), O 1s (b), C 1s (c), Cl 2p (d), S 2p (e), and Au 4f (f) XPS profiles of CEPS before (non‐contaminated and contaminated) and after photocatalytic decontamination over bare TiO_2_/Ti and Au_20s_/TiO_2_/Ti under UVC light condition.

**Chemical states of surface residues examined by XPS**. XPS characterization was used for selected samples of TiO_2_/Ti and Au‐loaded (20 s) TiO_2_/Ti catalysts (refer to Figure 5). It is worth noting that the catalysts underwent a drying process prior to their introduction into the ultrahigh vacuum chamber for analysis, which may have caused some surface alterations. Nevertheless, qualitative changes were observed in the samples both before and after the photocatalytic decontamination process.

For Ti 2p XPS profiles, the Ti 2p_3/2_ and Ti 2p_1/2_ peaks were consistently observed at binding energies (BEs) of 458.8 eV and 464.5 eV, featuring a spin‐orbit (S−O) splitting energy of 5.7 eV, characteristic of Ti(IV) in TiO_2_.[^32^, ^34^, ^42^] Notably, there was no significant change in the BE positions observed before and after CEPS contamination. However, upon UVC photocatalytic decontamination, a substantial reduction in the Ti 2p signals was noted for both TiO_2_/Ti and Au_20s_/TiO_2_/Ti, likely due to the accumulation of photo‐decontaminated residues, as discussed in Figures 1 and 2. Regarding the O 1s XPS profiles of TiO_2_/Ti and Au_20s_/TiO_2_/Ti before and after CEPS contamination, two broad peaks were consistently identified at 529.8 eV and 531.7 eV. These were attributed to metal oxide and surface oxide species, respectively.[^32^, ^42^] Following UVC photocatalytic decontamination, the O 1s peak shifted to a common center at approximately 530.8 eV, reflecting a modification in surface oxygen states. The C 1s peak underwent a substantial change before and after UVC photocatalytic decontamination, with a marked increase in intensity observed after photocatalytic decontamination due to the presence of photo‐decontaminated residues on the surface. The BE position also shifted slightly lower, reflecting the accumulation of broken residues. Similarly, the Cl 2p peaks were enhanced after photocatalytic decontamination, with Cl 2p_3/2_ and Cl 2p_1/2_ located at 198.4 eV and 200 eV, featuring a S−O separation of 1.6 eV, attributed to the C−Cl bond in CEPS.^[28]^ Following photocatalytic decontamination, the BE position was shifted to a higher BE by 1.0 eV. The S 2p peaks exhibited more drastic changes before and after photocatalytic decontamination. In non‐contaminated samples, no S 2p signal was detected, as expected. However, after contamination, S 2p_3/2_ and S 2p_1/2_ peaks appeared at 162.3 eV and 163.5 eV, with a S−O separation of 1.2 eV, associated with the S−C bond in CEPS.^[28]^ Subsequent to photocatalytic decontamination, the S 2p signal substantially increased, similar to the C 1s and Cl 2p peaks. Additionally, new prominent S 2p_3/2_ and S 2p_1/2_ peaks emerged at 167.1 eV and 168.3 eV, attributed to the presence of sulfoxide (S=O) groups.^[45]^ The formation of a product with a sulfoxide group was corroborated by the GC‐mass profile. In the case of Au_20s_/TiO_2_/Ti, before and after CEPS contamination, Au 4f_7/2_ and 4f_5/2_ XPS peaks were consistently located at 84.0 eV and 87.7 eV, featuring a S−O splitting energy of 3.7 eV, characteristic of metallic Au.^[46]^ However, following UVC contamination, the Au 4 f signal nearly disappeared due to the presence of thick photo‐decontaminated residues on the surface. We did not conduct additional characterization on the Cu/TiO_2_/Ti and Pt/TiO_2_/Ti samples, as they were solely utilized to demonstrate similar diverse product results. Our emphasis has been on exploring the diversity of products and the role of an overlayer metal, specifically Au.

**Further insight into the photocatalytic decontamination mechanism**. As previously discussed, the reaction mechanism for each product has been examined. Further extend photoexcitation followed by decomposition, which was proposed after analyzing the photodecomposition products under specific experimental conditions, taking into account factors such as UV light wavelength and the presence of air. When operating in the presence of air, which contains O_2_, the role of oxygen within the proposed mechanism was considered. CEPS was excited through both direct photoexcitation and an indirect charge‐transfer excitation process (involving TiO_2_/Ti), depending on the wavelength of light. In the presence of UVC (200–290 nm) and UVB (290–320 nm) light, it was anticipated that both CEPS and TiO_2_/Ti would absorb the photons, leading to the generation of electrons and holes in the conduction and valence bands, respectively. Under intense UV light, the chemical bonds of CEPS were expected to undergo direct photoexcitation, resulting in the formation of diverse radical species, as elaborated below.[^31^, ^32^, ^42^]

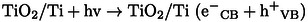




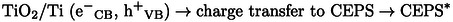




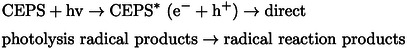




Several end products contained oxygen atoms within their molecular structures. Given that CEPS lacks oxygen in its molecular structure, the sole anticipated source of oxygen (including H_2_O) was from the surrounding air. In the presence of O_2_, the photoexcited electron can be captured by O_2_ to generate superoxide ⋅O_2_^−^ species, following the reaction O_2_+e^−^→⋅O_2_^−^. Singlet oxygen (^1^O_2_) may also form directly through UV absorption, as in the reaction O_2_→^1^O_2_, as previously documented.^[27]^ Under UVC conditions, the creation of ozone cannot be ruled out, potentially occurring via O_2_→2O and O_2_+O→O_3_, with ozone participating in subsequent oxidation reactions. These reactive oxygen species are anticipated to engage in oxidation reactions leading to the formation of products such as sulfoxides, sulfones, and carboxylic acids. Additionally, other potential reactions generating reactive species include H_2_O_2_ and ⋅OH radicals, as described below.[^31^, ^32^, ^42^]

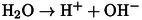




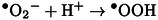




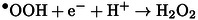




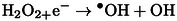




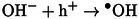




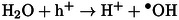




**Figure 6:**
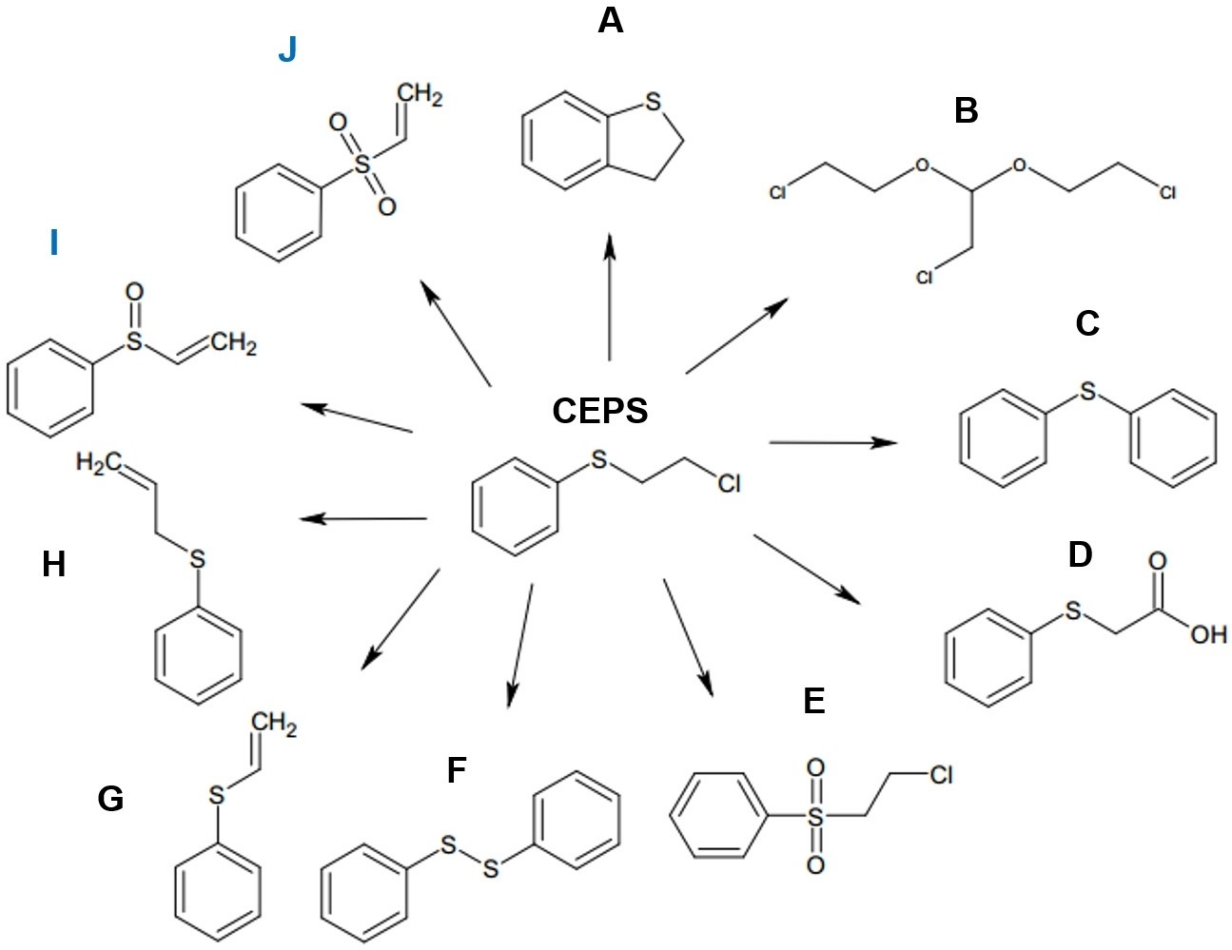
Secondary byproducts formed under UV light photocatalytic decontamination of CEPS. I and J were only produced under UVB light conditions.

These species play a role in decomposition, oxidation, and hydrolysis reactions, as discussed earlier in the context of the mechanism for each secondary byproduct. In conclusion, the interaction of different reactive species and the cleavage of bonds through direct photolysis collectively lead to the generation of a diverse array of secondary byproducts (refer to Figure 6). This underscores the importance of careful consideration to ensure the safety of treatment procedures. While the decomposition products seem to be safer than those of the CEPS, it is essential to acknowledge that they still possess some toxicities. Therefore, a post‐treatment step becomes imperative in UV decontamination methods.

## 3 Conclusions

In summary, this study investigated the photocatalytic decontamination of the CWA simulant molecule CEPS on metal (Au, Cu, and Pt)‐loaded TiO_2_/Ti sheets under both UVC and UVB conditions. The TiO_2_/Ti substrate featured rutile TiO_2_ grown directly on metallic Ti support. Under UVC conditions, at least eight distinct secondary byproducts were identified, with four major products including (phenylthio)acetic acid (C_8_H_8_O_2_S), diphenyl disulfide (C_12_H_10_S_2_), phenyl vinyl sulfide (C_8_H_8_S), and allyl phenyl sulfide (C_9_H_10_S). Notably, under UVC conditions, bare TiO_2_/Ti exhibited higher activity in comparison to Au/TiO_2_/Ti. Conversely, under UVB conditions, Au/TiO_2_/Ti demonstrated significantly enhanced activity when compared to TiO_2_/Ti, underscoring the vital role played by the overlayer Au. It was observed that the cleavage of C−Cl bonds appeared to be facilitated by an interaction between Au and Cl under UVB conditions. In the photodecomposition experiments involving M/TiO_2_/Ti (M=Au, Cu, and Pt) under UVC conditions, the overlayer Pt exhibited higher activity compared to the other overlayer metals. Under UVB conditions, five secondary byproducts were identified with a main (phenylthio)acetic acid, and two of them, featuring sulfone and sulfoxide functional groups, were newly observed. These products were generated through oxidation reactions involving sulfide in the presence of reactive oxygen species such as ⋅O_2_^−^ and singlet oxygen. In short, UVC demonstrated greater efficiency compared to UVB, generating more by‐products due to increased bond breakages. Au/TiO_2_ proved to be more efficient than bare TiO_2_ under UVB irradiation. However, UVC exhibited the highest efficiency over bare TiO_2_, with minimal effects observed from the overlaid metal. These demonstration tests offer valuable insights into the advancement of UV light photocatalytic decontamination methods. They also contribute to a deeper understanding of wavelength‐dependent photocatalytic reaction pathways, the formation of secondary byproducts after photocatalytic decontamination, and the strategies for addressing these byproducts based on real treatment conditions.

## Conflict of interests

The authors report no declarations of interest.

4

## Data Availability

Data will be made available upon a reasonable request.

## References

[1] Y. J. Jang , K. Kim , O. G. Tsay , D. A. Atwood , D. G. Churchill , Chem. Rev. 2015, 115, PR1–PR76. 26654832 10.1021/acs.chemrev.5b00402

[2] S. S. Talmage , A. P. Watson , V. Hauschild , N. B. Munro , J. King , Curr. Org. Chem. 2007, 11, 285–298.

[3] E. Oheix , E. Gravel , E. Doris , Chem. Eur. J. 2021, 27, 54–68. 32876358 10.1002/chem.202003665

[4] Y. C. Yang , J. A. Baker , J. R. Ward , Chem. Rev. 1992, 92, 1729–1743.

[5] K. Vellingiri , L. Philip , K.-H. Kim , Coord. Chem. Rev. 2017, 353, 159–179.

[6] G. W. Wagner , Y. C. Yang , Ind. Eng. Chem. Res. 2002, 41, 1925–1928.

[7] Y. Liu , A. J. Howarth , N. A. Vermeulen , S.-Y. Moon , J. T. Hupp , O. K. Farha , Coord. Chem. Rev. 2017, 346, 101–111.

[8] B. Picard , I. Chataigner , J. Maddaluno , J. Legros , Org. Biomol. Chem. 2019, 17, 6528–6537. 31215586 10.1039/c9ob00802k

[9] J. Nawała , P. Jóźwik , S. Popiel , Int. J. Environ. Sci. 2019, 16, 3899–3912.

[10] S. Chauhan , S. Chauhan , R. D'Cruz , S. Faruqi , K. K. Singh , S. Varma , M. Singh , V. Karthik , Environ. Toxicol. Pharmacol. 2008, 26, 113–122. 21783898 10.1016/j.etap.2008.03.003

[11] S.-Y. Lee , D.-I. Jang , D.-Y. Kim , K.-J. Yee , H.-Q. Nguyen , J. Kim , Y. Sohn , H. Jung , J. Photochem. Photobiol. A 2021, 406, 112989.

[12] L. Zhang , C. Sun , S.-J. Xiao , Q.-G. Tan , G.-P. Yang , J.-Q. Fan , Y.-T. Luo , R.-P. Liang , J.-D. Qiu , ACS Appl. Nano Mater. 2023, 6, 17083–17091.

[13] M. Šťastný , V. Štengl , J. Henych , J. Tolasz , M. Kormunda , J. Ederer , G. Issa , P. Janoš , RSC Adv. 2020, 10, 19542–19552. 35515455 10.1039/d0ra00944j PMC9054062

[14] V. G. Snider , C. L. Hill , J. Hazard. Mater. 2023, 442, 130015. 36166906 10.1016/j.jhazmat.2022.130015

[15] H. Zhao , C.-A. Tao , S. Zhao , X. Zou , F. Wang , J. Wang , ACS Appl. Mater. Interfaces 2023, 15, 3297–3306. 36608147 10.1021/acsami.2c18126

[16] H. Wang , J. Zhong , C. Zhao , X. Guo , Y. Zhao , J. Environ. Chem. Eng. 2021, 9, 105938.

[17] C. Zhou , B. Yuan , S. Zhang , G. Yang , L. Lu , H. Li , C. Tao , ACS Appl. Mater. Interfaces 2022, 14, 23383–23391. 10.1021/acsami.2c02401 35549001

[18] D. Li , H. Xi , Z. Wang , D. Wang , Z. Li , S. Zhao , ChemistrySelect 2019, 4, 13307–13312.

[19] W. Gordon , A. Balboa , S. Giles , A. Epshteyn , O. Ávalos-Ovando , A. Govorov , M. McEntee , O. Baturina , Crystals 2021, 11, 659.

[20] M. Sadeghi , S. Yekta , H. Ghaedi , Appl. Surf. Sci. 2017, 400, 471–480.

[21] Y. Hao , E. K. Papazyan , Y. Ba , Y. Liu , ACS Catal. 2022, 12, 363–371.

[22] A. Kiani , K. Dastafkan , J. Colloid Interface Sci. 2016, 478, 271–279. 27309947 10.1016/j.jcis.2016.06.025

[23] R. Wang , Z. Li , X. Li , P. Guo , H. Wang , X. Guo , J. Zhong , J. Water Process Eng. 2023, 53, 103647.

[24] Z. Zhu , J. Yu , Environ. Res. 2021, 197, 111082. 33812875 10.1016/j.envres.2021.111082

[25] S. L. Giles , A. M. Kastl , A. P. Purdy , A. C. Leff , D. C. Ratchford , W. A. Maza , O. A. Baturina , ACS Appl. Mater. Interfaces 2022, 14, 9655–9666. 35134290 10.1021/acsami.1c18180

[26] H. Wang , G. W. Wagner , A. X. Lu , D. L. Nguyen , J. H. Buchanan , P. M. McNutt , C. J. Karwacki , ACS Appl. Mater. Interfaces 2018, 10, 18771–18777. 29766717 10.1021/acsami.8b04576

[27] Y. Yang , J. Yin , F. Tao , Y. Zhou , L. Zhang , Y. Zhong , Y. A. Wang , RSC Adv. 2022, 12, 20251–20258. 35919596 10.1039/d2ra01821g PMC9277536

[28] L. Liu , K.-A. Min , M. Tayyab , B. Han , C.-H. Lee , Environ. Adv. 2022, 9, 100255.

[29] M. Lou , A. Bayles , H. O. Everitt , N. J. Halas , Nano Lett. 2022, 22, 7699–7705. 36073653 10.1021/acs.nanolett.2c03188

[30] T. Wu , F. Qiu , R. Xu , Q. Zhao , L. Guo , D. Chen , C. Li , X. Jiao , ACS Appl. Mater. Interfaces 2023, 15, 1265–1275. 36594244 10.1021/acsami.2c19039

[31] T. Kim , J. H. Yang , S. J. Park , H.-Q. Nguyen , J. Kim , K.-J. Yee , H. Jung , J.-G. Kang , Y. Sohn , Appl. Catal. B 2021, 284, 119623.

[32] J. H. Yang , S. J. Park , S.-M. Hong , J. Kim , K.-J. Yee , H. Jung , Y. Sohn , J. Ind. Eng. Chem. 2021, 100, 75–91.

[33] R. Tsyshevsky , M. McEntee , E. M. Durke , C. Karwacki , M. M. Kuklja , ACS Appl. Mater. Interfaces 2021, 13, 696–705. 33350299 10.1021/acsami.0c19261

[34] M. M. Khan , S. A. Ansari , D. Pradhan , M. O. Ansari , D. H. Han , J. Lee , M. H. Cho , J. Mater. Chem. A. 2014, 2, 637–644.

[35] D. L. J. Clive , S. P. Fletcher , D. Liu , J. Org. Chem. 2004, 69, 3282–3293. 15132533 10.1021/jo030364k

[36] M. S. Ahmed , D. S. Mannel , T. W. Root , S. S. Stahl , Org. Process Res. Dev. 2017, 21, 1388–1393.

[37] M. Assis , A. F. Gouveia , L. K. Ribeiro , M. A. Ponce , M. S. Churio , O. N. Oliveira , L. H. Mascaro , E. Longo , R. Llusar , E. Guillamón , J. Andrés , Appl. Catal. A 2023, 652, 119038.

[38] D. H. Dethe , A. Srivastava , B. D. Dherange , B. V. Kumar , Adv. Synth. Catal. 2018, 360, 3020–3025.

[39] K. Tada , K. Sakata , Y. Kitagawa , T. Kawakami , S. Yamanaka , M. Okumura , Chem. Phys. Lett. 2013, 579, 94–99.

[40] P. Ríos , A. Rodríguez , S. Conejero , Chem. Commun. 2020, 56, 5333–5349. 10.1039/d0cc01438a 32373864

[41] T. A. Baker , C. M. Friend , E. Kaxiras , J. Am. Chem. Soc. 2008, 130, 3720–3721. 18314988 10.1021/ja7109234

[42] J. H. Yang , S. J. Park , C. K. Rhee , Y. Sohn , Nanomater. 2020, 10, 1909. 10.3390/nano10101909 PMC7600856 32987906

[43] J. Wu , S. Ma , Z. Hu , J. Wang , J. Wang , Y. Wu , Crystals 2022, 12, 1789.

[44] O. Frank , M. Zukalova , B. Laskova , J. Kürti , J. Koltai , L. Kavan , Phys. Chem. Chem. Phys. 2012, 14, 14567–14572. 23014450 10.1039/c2cp42763j

[45] W.-S. Jeon , C. H. Kim , J.-H. Wee , J. H. Kim , Y. A. Kim , C.-M. Yang , Appl. Surf. Sci. 2021, 558, 149867.

[46] Y. J. Kim , J. Y. Maeng , S. Y. Hwang , J. H. Yang , I. Yoon , C. W. Myung , C. K. Rhee , Y. Sohn , Nano Energy 2023, 118, 108995.

